# Age-related deficits in skeletal muscle recovery following disuse are associated with neuromuscular junction instability and ER stress, not impaired protein synthesis

**DOI:** 10.18632/aging.100879

**Published:** 2016-01-29

**Authors:** Leslie M. Baehr, Daniel W.D. West, George Marcotte, Andrea G. Marshall, Luis Gustavo De Sousa, Keith Baar, Sue C. Bodine

**Affiliations:** ^1^ VA Northern California Health Care System, Mather, CA 95655, USA; ^2^ Department of Physiology and Membrane Biology, University of California Davis, Davis, CA 95616, USA; ^3^ Department of Neurobiology, Physiology and Behavior, University of California Davis, Davis, CA 95616, USA

**Keywords:** aging, hindlimb unloading, anabolic resistance, ubiquitin proteasome system, autophagy

## Abstract

Age-related loss of muscle mass and strength can be accelerated by impaired recovery of muscle mass following a transient atrophic stimulus. The aim of this study was to identify the mechanisms underlying the attenuated recovery of muscle mass and strength in old rats following disuse-induced atrophy. Adult (9 month) and old (29 month) male F344BN rats underwent hindlimb unloading (HU) followed by reloading. HU induced significant atrophy of the hindlimb muscles in both adult (17-38%) and old (8-29%) rats, but only the adult rats exhibited full recovery of muscle mass and strength upon reloading. Upon reloading, total RNA and protein synthesis increased to a similar extent in adult and old muscles. At baseline and upon reloading, however, proteasome-mediated degradation was suppressed leading to an accumulation of ubiquitin-tagged proteins and p62. Further, ER stress, as measured by CHOP expression, was elevated at baseline and upon reloading in old rats. Analysis of mRNA expression revealed increases in HDAC4, Runx1, myogenin, Gadd45a, and the AChRs in old rats, suggesting neuromuscular junction instability/denervation. Collectively, our data suggests that with aging, impaired neuromuscular transmission and deficits in the proteostasis network contribute to defects in muscle fiber remodeling and functional recovery of muscle mass and strength.

## INTRODUCTION

Aging is associated with a progressive decline in muscle mass and strength, a condition known as sarcopenia. Sarcopenia is a major contributing factor to increased frailty and the loss of mobility seen in older adults [1]. Sarcopenia is also increasingly recognized as a prognostic indicator for patients who are likely to experience postoperative complications following organ transplants, cancer resection surgery, and for chemotherapy toxicity risk [2-5]. Although variable between individuals, it has been estimated that loss of skeletal muscle occurs at a rate of 1-2% per year after the age of 50, with losses in strength occurring more rapidly, such that older muscles become disproportionately weak [6]. Furthermore, the progression of sarcopenia can be accelerated by conditions that induce muscle atrophy, such as disuse, malnutrition, and diabetes. Transient periods of atrophy are particularly devastating to the elderly due to an age-related impairment in muscle recovery. This poor recovery of muscle mass in older individuals can result in longer hospital stays, potential long-term disability, reduced quality of life, and ultimately increased mortality risk [7]. Given the growing size of the elderly population and the tendency for this group to experience prolonged periods of inactivity, there is a substantial need to better understand the cellular and molecular mechanisms that regulate muscle mass and function with age.

Skeletal muscle is a plastic tissue that can modify its size and functional characteristics in response to a variety of signals. One potent signal for a loss of muscle mass is disuse, which can be caused by unloading or decreased neural activation [8]. The consequences of muscle disuse have been extensively studied in humans using models of limb immobilization and bed rest, and in rodents using hindlimb unloading (HU). In all three models, significant decreases in muscle size and strength have been observed after fourteen days [9-12], with some studies reporting that short-term disuse (≤7 days) can also cause significant loss of muscle mass and function [13-15]. Aging does not appear to exacerbate the atrophy process during disuse, as the drop in mass/cross-sectional area (CSA) is similar, or less, in older muscles compared to young [9, 16-19]. However, aging does have a major effect on the recovery of muscle mass following disuse. Upon return to normal weight bearing activity, losses in muscle mass and strength caused by unloading or inactivity can be reversed, but recovery in older individuals is often incomplete or delayed compared to young individuals [9, 10]. In young and old men undergoing 4 weeks of retraining after two weeks of knee immobilization, only the young men experienced full restoration of their muscle fiber CSA and mechanical function [10]. Similar observations have been made in aged rats, as muscle mass does not fully recover after forty days of reloading following eight days of hindlimb casting [20] or after two weeks of reloading following two weeks of HU [17].

Although it has been well documented that recovery is impaired in aged muscle, the mechanisms responsible for this attenuated growth response remain unclear. Age-related deficits in the activation of muscle protein synthesis (MPS) have been reported following ingestion of essential amino acids [21] and acute exercise [22, 23], suggesting that aging leads to an impaired response to anabolic stimuli, termed anabolic resistance. This diminished protein synthetic response may be due to reduced activation of the Akt/mTORC1 signaling pathway, which controls translational activity and is important in muscle growth [24]. There are reports [21, 22] that older individuals completely lack or exhibit blunted phosphorylation of S6K1 and 4EBP1 following resistance exercise compared to young individuals, while similar age-related deficits have been reported in aged rats following a single bout of contractile activity [25]. A reduction or delay in the activation of the Akt/mTORC1 pathway has also been linked to the attenuated muscle hypertrophy observed in older rats in response to synergist ablation [19, 26]. Beyond the effect on translational activity, recent microarray analysis of plantaris muscles from adult and old mice that were subjected to synergist ablation revealed that the blunted growth response in old mice was the result of impaired ribosome biogenesis, or translational capacity [27]. Together, these data suggest that the ability to increase translation activity and capacity is compromised in aged skeletal muscle.

The recovery of muscle mass following disuse-induced atrophy also requires the activation of protein degradation pathways to remove proteins associated with atrophy and to recycle amino acids from proteins rapidly synthesized upon reloading, but not incorporated into the myofibrils [28-30]. In skeletal muscle, the two major pathways responsible for the majority of protein breakdown are the ubiquitin proteasome system (UPS) and autophagy [31, 32]. With normal aging, there is evidence that overall proteolytic activity decreases, leading to an accumulation of misfolded and modified proteins that can have severe pathological consequences [33]. However, little is known about whether aging impairs the ability of skeletal muscle to activate degradation pathways during the remodeling and growth of muscle fibers. A loss in proteolytic capacity could potentially impair or delay the muscle remodeling process triggered upon reloading. Altogether, concomitant activation of protein synthesis and protein degradation pathways is likely to be imperative in the recovery of muscle mass following an atrophy-inducing event. Thus, the purpose of this study was to determine whether reductions in protein synthesis and/or inadequate protein degradation contribute to the age-related impairment of muscle recovery and function following disuse-induced muscle atrophy.

## RESULTS

### Hindlimb unloading results in diminished force production in Old

**Figure 1:**
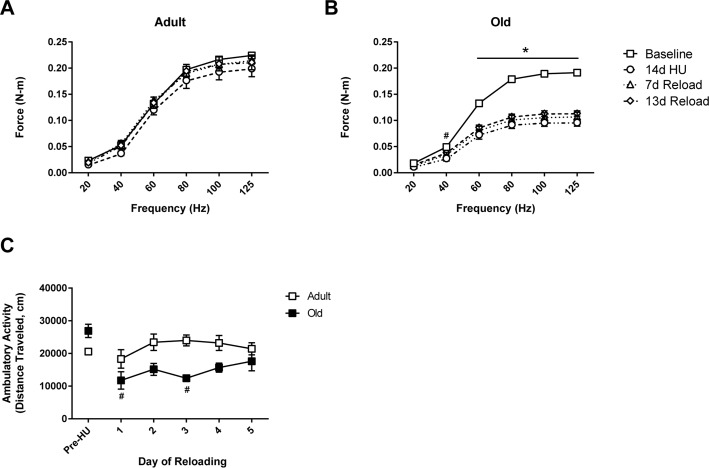
Force output and activity measures in adult and old rats *In vivo* isometric force production at frequencies ranging from 20-125 Hz was measured in **(A)** adult (9 mo) and **(B)** old (29 mo) rats prior to hindlimb unloading (HU) (baseline, open squares), after 14 days of HU (open circles), and then after 7 (open triangles) and 13 days of reloading (open diamonds). n=6/group. Values are mean ± SEM, *p<0.05 vs old baseline value, ^#^p<0.05 for old 14d HU value vs old baseline value at 40 Hz. **(C)** Normal cage activity of adult (open squares, n=4) and old (filled squares, n=5) rats was recorded during the dark cycle for six days prior to HU and for the first five days of reloading following 14 days of HU. Values are mean ± SEM, ^#^p<0.05 vs old control.

To determine the effect of age on the functional capacity of hindlimb muscles in response to unloading and reloading, *in vivo* isometric torque was measured at baseline (control), after 14 days of HU, and at days 7 and 13 of reloading (Fig. 1). At baseline, significant differences in maximum torque were found between Adult and Old (−14% in Old vs. Adult at 125 Hz, p<0.05). Following 14 days of HU, maximum torque decreased by 17% (p>0.05) in Adult, and 39% (p<0.05) in Old. Upon reloading, the torque-frequency relationship returned to control values in Adult after 7 days (Fig. 1A). In contrast, torque production in Old showed no significant improvement relative to the 14d unloading values during the 14 day reloading period (Fig. 1B).

### Home cage activity is reduced in Old in early recovery

To understand whether the lack of recovery in force production was related to a decrease in the use of the hindlimb muscles in Old, home cage activity was monitored during the initial days of reloading and compared to the activity levels measured prior to unloading. As shown in Figure 1C, home cage activity was similar in Adult and Old prior to unloading. Upon reloading, the activity levels of Adult were similar to pre-unloading values, while Old had a significant decrease in activity levels for the first three days of reloading. The difference in home cage activity in Old was equivalent to 13.8 meters/day. By day five of reloading, home cage activity levels were similar between Adult and Old.

### Recovery of muscle mass is impaired in Old following 14 days of hindlimb unloading

**Figure 2:**
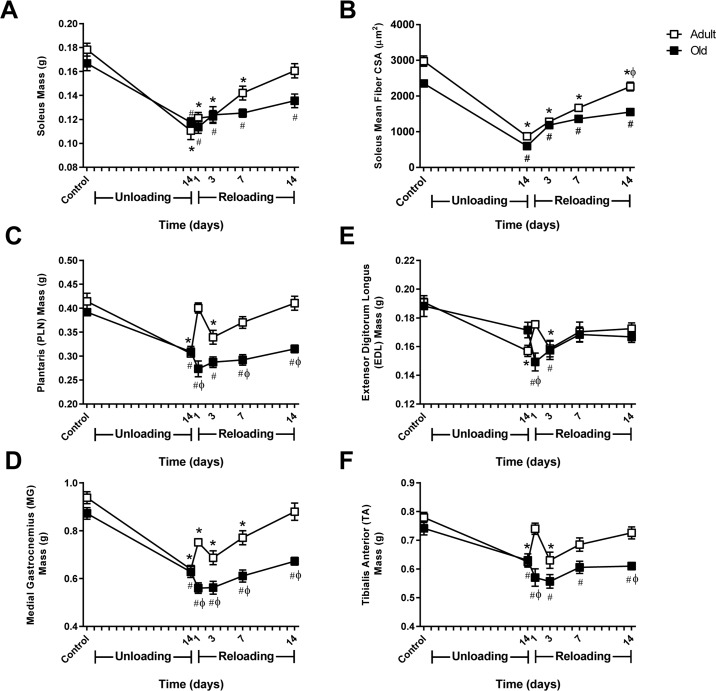
Comparison of muscle mass and soleus fiber cross-sectional area changes in adult and old rats subjected to hindlimb unloading (HU) and reloading Adult (9 mo) and old (29 mo) male rats underwent HU for 14 days or underwent HU for 14 days and then were allowed to resume normal weight bearing activity for either 1, 3, 7, or 14 days. Average masses of the soleus **(A)**, plantaris **(C)**, medial gastrocnemius **(D)**, extensor digitorum longus **(E)**, and tibialis anterior **(F)** muscles of adult (open squares) and old (filled squares) rats at the various HU and reloading time points (n=6-7/group). **(B)** Changes in fiber cross-sectional area (CSA) were measured in the soleus of adult (open squares) and old (filled squares) rats after 14 days of HU and after 3, 7, and 14 days of reloading. Fiber CSA was determined from laminin-stained cross sections (n=5-6/group). Values are mean ± SEM, *p<0.05 vs adult control, ^#^p<0.05 vs old control, ^φ^p<0.05 vs adult at same time point.

Muscle mass was monitored in both ankle extensor (soleus, MG, and PLN) and flexor (TA, EDL) muscles over 14 days of unloading and 14 days of reloading (Fig. 2A, C-F). At baseline, muscle mass tended to be higher in Adult compared to Old, although the differences in mass were not statistically significant for any of the muscles. Significant muscle atrophy occurred in all muscles except the EDL after 14 days of HU, with the absolute muscle loss being greater in Adult than Old. In general, loss of muscle mass was more severe in the extensors compared to the flexors for both age groups.

Upon reloading, Adult showed immediate recovery of muscle mass, while recovery in Old was delayed. In Adult, muscle mass increased progressively after reloading and returned to control levels by day 14 (Fig. 2A, C-F). In contrast, Old showed continued loss of mass in all muscles at day one of reloading, with the mass of the TA continuing to drop over the first three days of reloading. Overall, recovery of muscle mass in Old occurred at a slower rate resulting in only partial recovery by day 14.

Measurement of fiber cross-sectional area (CSA) in the soleus showed a similar pattern of recovery as observed in muscle mass (Fig. 2B). Soleus muscle fibers increased in size during reloading; however recovery was significantly better in Adult compared to Old.

### The protein synthesis response to reloading is not impaired in Old

**Figure 3:**
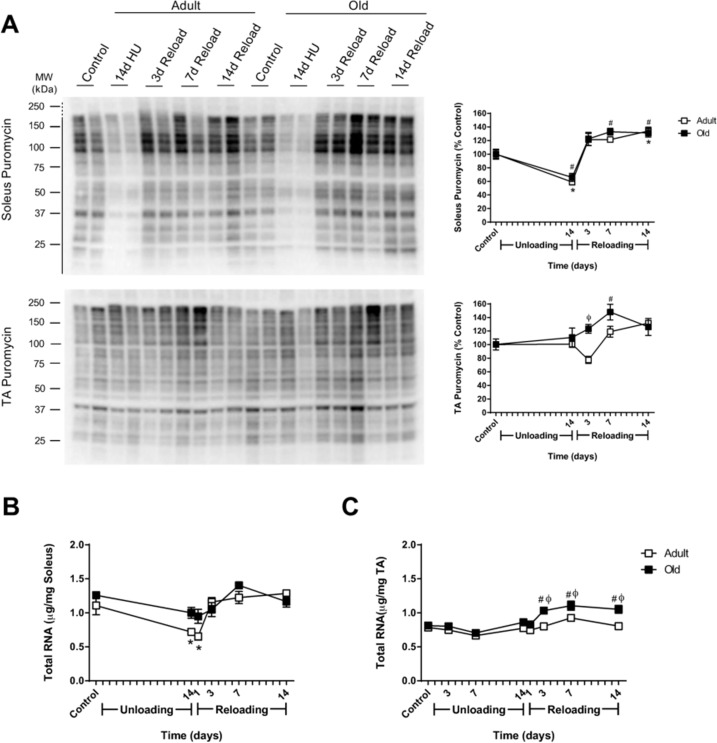
Effect of hindlimb unloading (HU) and reloading on muscle protein synthesis and total RNA in adult and old rats **(A)** Using the SUnSET method, protein synthesis was measured in the soleus and tibialis anterior (TA) muscles of adult (9 mo, open squares) and old (29 mo, filled squares) rats after 14 days of HU and after 3, 7, and 14 days of reloading. Total protein, determined by stain-free imaging of the PVDF membrane, was used to normalize protein expression. Puromycin values are expressed as a percentage of each age-matched control group (n=6-7/group). Total RNA (μg/mg muscle) was measured in the soleus **(B)** and TA **(C)** muscles of adult (open squares) and old (filled squares) rats after 14 days of HU and after 1, 3, 7, and 14 days of reloading (n=6/group). Values are mean ± SEM, *p<0.05 vs adult control, ^#^p<0.05 vs old control, ^φ^p<0.05 vs adult at same time point.

Previous reports have suggested that anabolic resistance is the primary cause of age-associated attenuation of muscle growth, and therefore, protein synthesis was measured using the SUnSET technique. The biochemical and molecular assessment of muscle regrowth was limited to the soleus, the primary ankle extensor muscle, and the TA, the primary ankle flexor muscle. In control rats, no differences in MPS were found with age in the soleus or TA muscles (data not shown). After 14 days of HU, MPS was significantly suppressed in the soleus muscle of both age groups, but was similar to control levels in Adult and Old TA muscles (Fig. 3A). Upon reloading, MPS increased in the soleus of both adult and old rats and remained elevated at day 14 of reloading (Fig. 3A). In the TA, slight differences were found between Adult and Old. In Adult TA, MPS decreased immediately upon reloading, but then increased to levels greater than baseline. In Old TA, MPS increased immediately upon reloading and remained higher than baseline for the 14 day reloading period. Taken as a whole, these results indicate that the MPS response to reloading was not attenuated in Old. Moreover, Old increased MPS even though they had a decrease in overall cage activity during the first 5 days of reloading.

### Activation of the Akt/mTORC1 pathway is similar between Adult and Old during reloading

To further characterize the MPS response to reloading, the phosphorylation and total protein levels of Akt, GSK3β, S6K1, and 4EBP1 were measured (Supplemental Fig. 1 and 2). In general, Akt and mTORC1 were activated in both the soleus and TA muscles upon reloading, and there were no major age-related differences.

### Old show greater increases in ribosome biogenesis markers in response to reloading

Since the rate of protein synthesis is determined by both the translational activity and capacity of a cell [34], and translational capacity is largely dependent on ribosomal number and availability, the ability to increase ribosome mass in response to reloading may affect rates of MPS, and ultimately recovery. Thus, total RNA was used as a measure of ribosomal RNA (rRNA) content given that 80-85% of total RNA is rRNA [35]. Age alone did not affect RNA levels, as total RNA/mg muscle was similar between Adult and Old control animals in both the soleus and TA (Fig. 3B and C). In Adult soleus, total RNA/mg muscle significantly decreased in response to 14 days of HU and returned to control levels upon reloading (Fig. 3B). In contrast, decreases in total RNA/mg muscle with HU, and increases with reloading, were more subtle in Old soleus and did not reach statistical significance. In the TA, only the Old had a significant increase in total RNA as a result of reloading, with significantly higher total RNA/mg muscle than age-matched controls and Adult muscles, between days three and 14 of reloading (Fig. 3B).

To further elucidate whether age impacts ribosome biogenesis in response to changes in muscle loading, expression of c-myelocytomatosis oncogene (c-Myc) and internal transcribed spacer 1 (ITS1, a readout of precursor rRNA) was measured. Following the HU and reloading periods, no significant changes in c-Myc or ITS1 expression were found in Adult soleus or TA muscles (Supplemental Fig. 3). Conversely, expression of c-Myc and ITS1 were both significantly upregulated at various times throughout the 14 day reloading period in Old TA muscle, which correlated with the increase in total RNA seen in this muscle (Supplemental Fig. 3).

### Hindlimb muscles of Old exhibit higher expression of genes associated with inactivity and/or denervation

**Figure 4:**
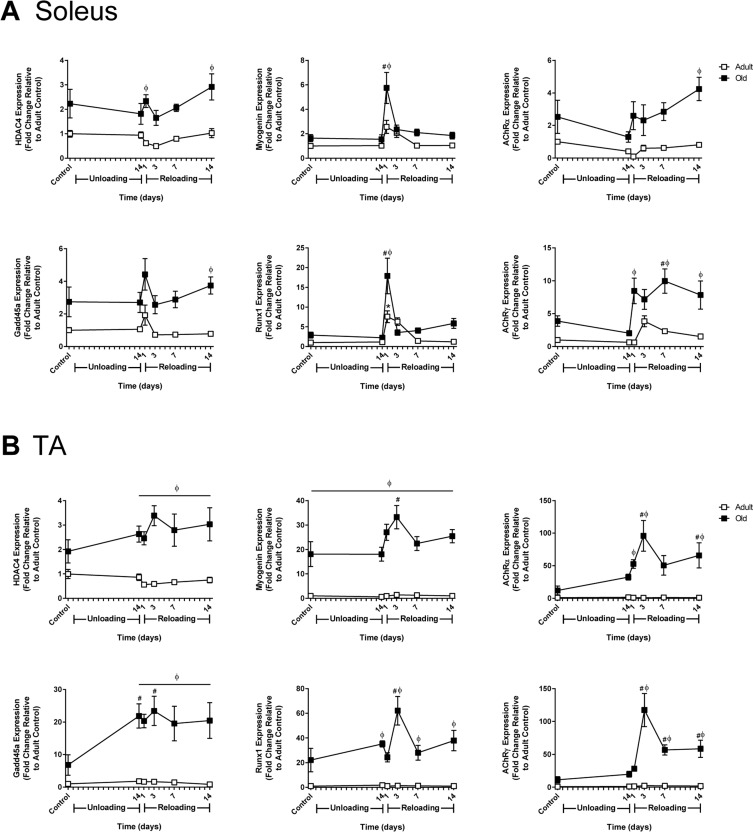
Effect of hindlimb unloading (HU) and reloading on gene expression markers of inactivity and/or denervation in adult and old rats mRNA expression (fold change relative to adult control) of HDAC4, myogenin, Gadd45a, Runx1, and acetylcholine receptor subunits alpha (AChRα) and gamma (AChRγ) were measured in the soleus **(A)** and tibialis anterior (TA) **(B)** muscles of adult (9 mo, open squares) and old (29 mo, filled squares) rats after 14 days of HU and after 1, 3, 7, and 14 days of reloading (n=5-6/group). Values are mean ± SEM, *p<0.05 vs adult control, ^#^p<0.05 vs old control, ^φ^p<0.05 vs adult at same time point.

The aging process is associated with instability of the neuromuscular junction and denervation as the result of motor neuron loss [36]. To assess denervation and neuromuscular junction instability at baseline and in response to unloading and reloading, the expression of HDAC4, myogenin, Runx1, Gadd45a, and the acetylcholine receptor subunits (AChR α, γ, δ, and ε) was measured in Adult and Old soleus and TA muscles (Fig. 4 and Supplemental Fig. 4). At baseline, there was a trend for HDAC4, myogenin, Runx1, and Gadd45a to be elevated in Old soleus and TA relative to Adult, with myogenin reaching significance in the Old TA (Fig. 4). Although not significant, all of the AChR subunits also tended to be elevated in Old at baseline (Fig. 4 and Supplemental Fig. 4).

In response to unloading or reloading, the only significant change in the expression level of the measured genes observed in Adult was an increase in Runx1 in the soleus at day 1 of reloading (Fig. 4 and Supplemental Fig. 4). In Old soleus and TA, no significant changes in gene expression were apparent after 14 days of unloading, with the exception of Gadd45a, which significantly increased in the TA. Upon reloading, significant changes in gene expression occurred in both the soleus and TA of Old, with greater increases in expression occurring in the TA compared to the soleus. A common pattern in all of the genes was a sharp rise in expression upon reloading that peaked at 3 days and then decreased. In Old TA, the expression of myogenin, HDAC4 and Gadd45a remained significantly elevated above Adult for the entire 14 day reloading period (Fig. 4B). Of the AChR subunits analyzed, AChRα and AChRγ showed the greatest increases in expression during reloading, with expression being significantly higher in the TA than the soleus (Fig. 4). The pattern of expression for the AChR subunits mimicked that of myogenin and HDAC4, which are thought to be upstream regulators of the AChR genes.

### Reloading does not increase activity of the ubiquitin proteasome system (UPS) in Old

Since the anabolic response to reloading was similar between Adult and Old, protein degradation pathways were examined to determine whether alterations in degradation could explain the attenuated growth response in the old rats. Given the importance of the ubiquitin proteasome pathway in the degradation of proteins within muscle, we measured ubiquitin levels and the activities of the three catalytic subunits (β1, β2, and β5) of the proteasome at baseline and in response to reloading.

**Figure 5:**
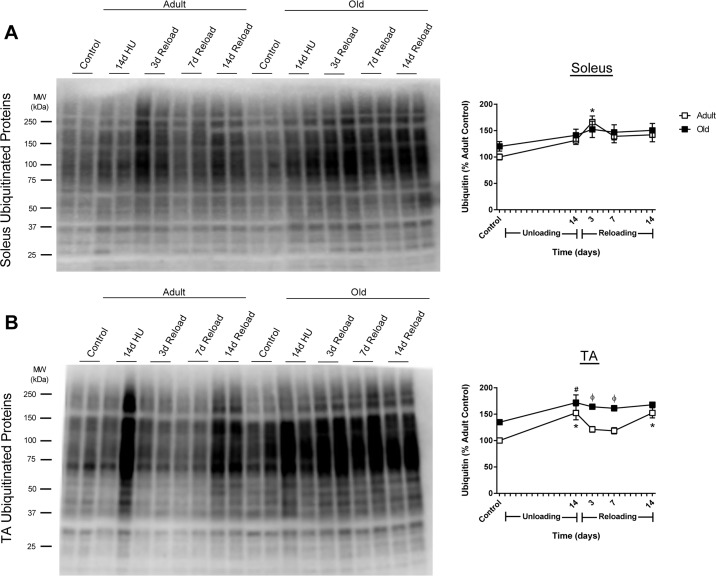
Total ubiquitin levels in adult and old rats after hindlimb unloading (HU) and reloading Representative Western blot and quantification of total ubiquitin levels in the soleus **(A)** and tibialis anterior (TA) **(B)** muscles of adult (9 mo, open squares) and old (29 mo, filled squares) rats after 14 days of HU and after 3, 7, and 14 days of reloading. Total protein, determined by stain-free imaging of the PVDF membrane, was used to normalize protein expression. Data are expressed as a percentage relative to the adult control group (n=5-6/group). Values are mean ± SEM, *p<0.05 vs adult control, ^#^p<0.05 vs old control, ^φ^p<0.05 vs adult at same time point.

At baseline, ubiquitin levels were similar between Adult and Old soleus, but tended to be higher in the TA of Old versus Adult (Fig. 5). In response to reloading, Adult and Old soleus had similar ubiquitin levels (Fig. 5A). In contrast, ubiquitin levels in Old TA were significant greater when compared to Adult TA during the first week of reloading (Fig. 5B).

**Figure 6:**
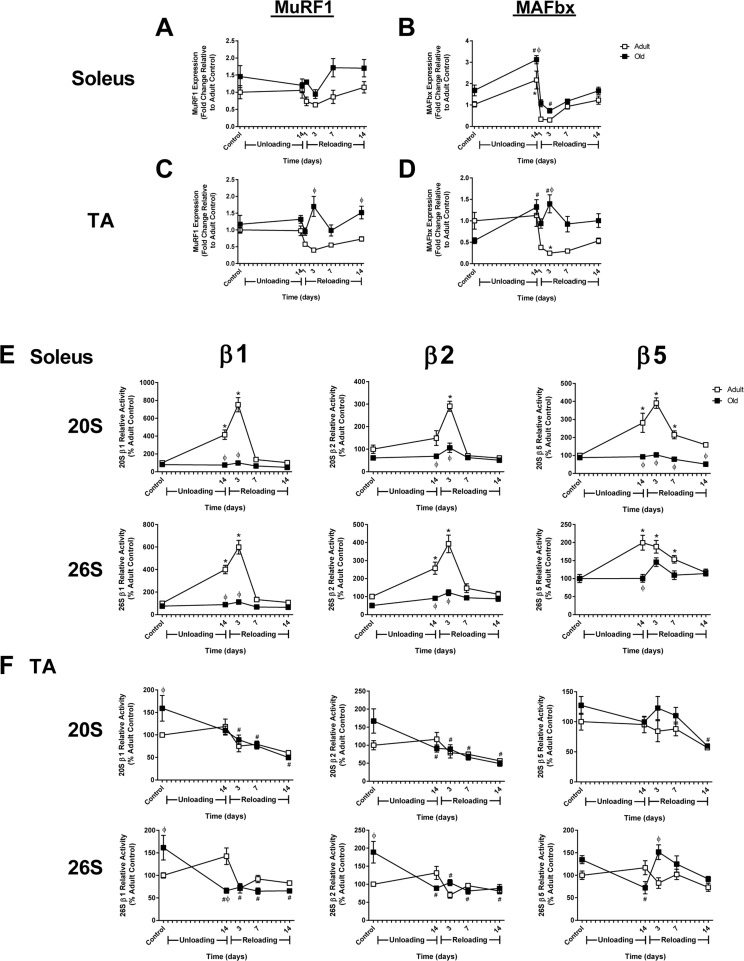
Changes in MuRF1 and MAFbx expression and proteasome activity in adult and old rats after hindlimb unloading (HU) and reloading mRNA expression of MuRF1 and MAFbx was assessed by quantitative PCR in the soleus **(A,B)** and tibialis anterior (TA) **(C,D)** muscles of adult (9 mo, open squares) and old (29 mo, filled squares) rats after 14 days of HU and following 1, 3, 7, and 14 days of reloading. Gene expression was normalized to tissue weight. Data are expressed as a fold change relative to the adult control group. Proteolytic activity of the β1, β2, and β5 subunits of the 20S and 26S proteasome was measured in the soleus **(E)** and TA **(F)** muscles of adult (open squares) and old (filled squares) rats after 14 days of HU and after 3, 7, and 14 days of reloading. Data are expressed as a percentage relative to the activity of the adult control group for each subunit (n=4-6/group). Values are mean ± SEM, *p<0.05 vs adult control, ^#^p<0.05 vs old control, ^φ^p<0.05 vs adult at same time point.

Measurement of baseline proteasome activity in the soleus revealed no significant differences between Adult and Old (Fig. 6E); however, in the TA, β1 and β2, but not β5, subunit activities were significantly elevated in Old (Fig. 6F). In response to unloading and reloading, proteasome activity was elevated in Adult soleus and remained elevated through 7 days of reloading (Fig. 6E). In contrast, proteasome activity was unaltered in Old soleus after unloading and upon reloading. In the TA, proteasome activity did not change significantly from baseline in Adult after 14 days of HU and reloading, while significant decreases in proteasome activity were observed in Old in response to unloading and reloading (Fig. 6F).

Expression of the E3 ligases, MuRF1 and MAFbx, was measured in Adult and Old muscles since these ligases are often associated with muscle atrophy and increased proteasome activity. In Adult, expression of MuRF1 and MAFbx was significantly suppressed upon reloading in the soleus, even though proteasome activity increased (Fig. 6A and B). In Adult TA, expression of both ligases was also suppressed upon reloading (Fig. 6C and D). In Old, MuRF1 was not suppressed in either the soleus or TA upon reloading, and in fact remained slightly above baseline. MAFbx expression varied across muscles upon reloading in Old, with expression remaining elevated in the TA, but suppressed in the soleus. Interestingly, the expression of MuRF1 and MAFbx in Old did not correlate with proteasome activity.

### Autophagy markers are higher at baseline and increase in response to reloading in Old

**Figure 7:**
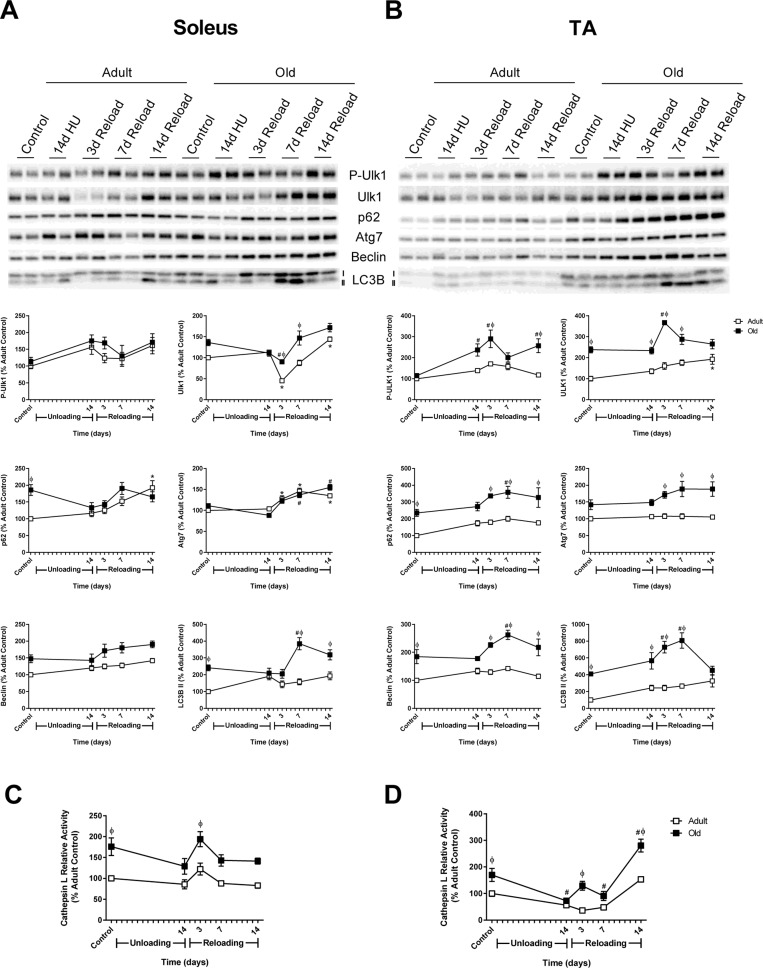
Autophagy-related changes in protein expression and cathepsin L activity during hindlimb unloading (HU) and reloading Expression of the autophagy-related proteins phospho- and total Ulk1, p62, Atg7, Beclin, and LC3B-II was measured by Western blot and quantified after 14 days of HU and following 3, 7, and 14 days of reloading in the soleus **(A)** and tibialis anterior (TA) **(B)** muscles of adult (9 mo, open squares) and old (29 mo, filled squares) rats. Total protein, determined by stain-free imaging of the PVDF membrane, was used to normalize protein expression. Data are expressed as a percentage relative to the adult control group for each protein (n=4-6/group). Cathepsin L activity was measured by fluorometric assay after 14 days of HU and following 3, 7, and 14 days of reloading in the soleus **(C)** and TA **(D)** muscles of adult (open squares) and old (filled squares) rats. Data are expressed as a percentage relative to the activity of the adult control group (n=5-6/group). Values are mean ± SEM, *p<0.05 vs adult control, ^#^p<0.05 vs old control, ^φ^p<0.05 vs adult at same time point.

Given that proteasome activity was not elevated in the old rats, we determined whether the other major degradation pathway in muscle, autophagy, was activated in response to unloading and/or reloading. Various markers of autophagy such as Atg7, Beclin, LC3B, and p62 were examined in the muscles of Adult and Old (Fig. 7A and B). In the soleus, expression levels of Atg7 and Beclin were similar between Adult and Old, increasing slightly upon reloading. In contrast, Atg7 and Beclin levels were significantly higher at baseline in Old TA and remained significantly higher throughout the reloading period. Both of the autophagosome-associated proteins, LC3B-II and the chaperone protein p62, were significantly elevated in Old soleus and TA at baseline (Fig. 7A and B). In response to unloading, the level of LC3B-II remained unchanged from baseline in Adult and Old. However, upon reloading, LC3B-II increased significantly in both Old soleus and TA muscles. Levels of p62 upon reloading were similar in the soleus between Adult and Old, but were significantly higher in Old TA relative to Adult. Another indicator of elevated autophagy is an increase in cathepsin L activity. Cathepsin L activity was elevated in the soleus and TA of Old, compared to Adult, at baseline and during the reloading period (Fig. 7C and D).

Collectively, these data suggested that autophagy was elevated in the TA, and possibly the soleus, of Old at baseline and in response to reloading. Initiation of autophagy is controlled by Atg1/Ulk1, which is inhibited through phosphorylation by mTORC1 [37]. Examination of Ulk1 phosphorylation revealed a significant increase in phosphorylation state in Old TA at 14 days of unloading and upon reloading (Fig. 7B). The increase in Ulk1 phosphorylation paralleled the phosphorylation status of S6K1 and reflected the increase in mTORC1 activity in both adult and old muscles upon reloading.

### Autophagic flux is higher in Old, but does not increase after seven days of reloading

**Figure 8:**
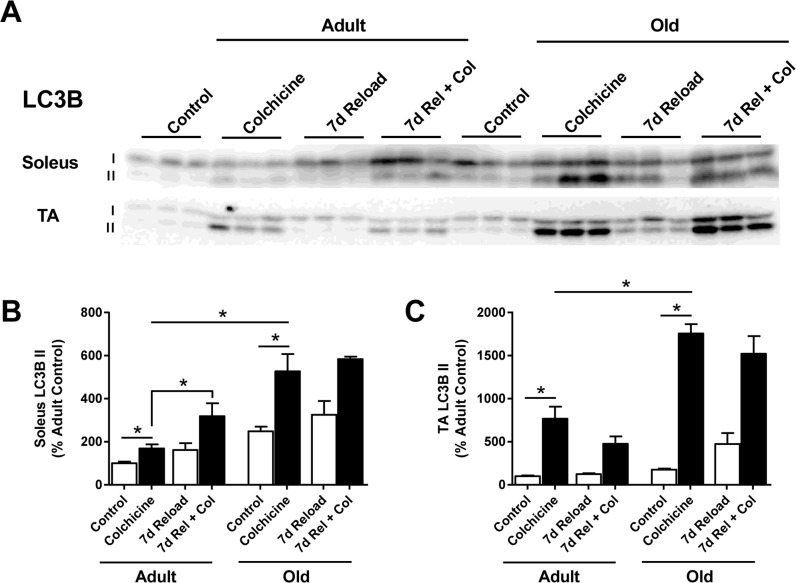
Effect of age and reloading on autophagic flux and p62 protein accumulation using the autophagy inhibitor colchicine Representative Western blots **(A)** and quantification of LC3B-II protein expression in the soleus **(B)** and tibialis anterior (TA) **(C)** muscles of control and 7 day reloaded adult (9 mo) and old (29 mo) rats treated with or without colchicine. Total protein, determined by stain-free imaging of the PVDF membrane, was used to normalize protein expression. Data are expressed as a percentage relative to the adult control group for each protein (n=3-6/group). Values are mean ± SEM, *p<0.05.

To determine whether autophagy was indeed elevated in Old, autophagic flux was measured *in vivo* at baseline and after 7 days of reloading using the autophagy inhibitor colchicine [38]. Two days of colchicine treatment in control rats caused a significant increase in LC3B-II protein levels in both Adult and Old soleus and TA muscles, indicating that autophagy was ongoing in these muscles (Fig. 8A-C). Interestingly, the increase in LC3B-II was significantly greater in Old compared to Adult, suggesting that the basal rate of autophagy was higher in the older muscles. Colchicine treatment also resulted in a significant increase in LC3B-II protein levels at 7 days of reloading. In Adult soleus, this increase in LC3B-II protein levels was significantly greater than the increase observed at baseline, indicating that autophagy was increased in this muscle upon reloading. However, in Old, the colchicine-induced increases in LC3B-II levels observed in the soleus and TA at 7 days of reloading were not greater than that seen at baseline, suggesting that autophagy was not upregulated in Old in response to reloading. Furthermore, greater p62 accumulation was observed in Old TA during the reloading period, suggesting that a block in autophagy activation may be occurring in the Old muscles during reloading (Supplemental Fig. 5).

### Increases in endoplasmic reticulum (ER) stress are greater in Old upon reloading

**Figure 9:**
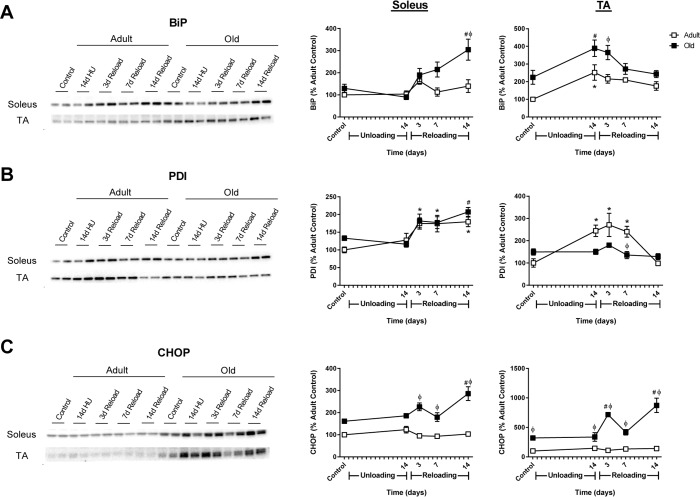
Markers of endoplasmic reticulum (ER) stress in adult and old rats during hindlimb unloading (HU) and reloading Representative Western blots and quantification of ER stress markers BiP **(A)**, PDI **(B)**, and CHOP **(C)** in the soleus and tibialis anterior (TA) muscles of adult (9 mo, open squares) and old (29 mo, filled squares) rats following 14 days of HU and after 3, 7, and 14 days of reloading. Total protein, determined by stain-free imaging of the PVDF membrane, was used to normalize protein expression. Data are expressed as a percentage relative to the adult control group for each protein (n=4-6/group). Values are mean ± SEM, *p<0.05 vs adult control, ^#^p<0.05 vs old control, ^φ^p<0.05 vs adult at same time point.

Despite the ability of old rats to increase ribosome biogenesis and MPS during reloading, the recovery of mass was significantly attenuated in the older muscles (Fig. 2). Periods of high growth in skeletal muscle can induce protein unfolding and ER stress; thus, the protein expression of the ER stress markers BiP, protein disulfide isomerase (PDI), and C/EBP homologous protein (CHOP) were measured in Adult and Old muscles. After 14 days of unloading, no changes in ER stress were observed in the soleus of Adult or Old rats (Fig. 9). Upon reloading, increases in PDI, but not BiP or CHOP were observed in Adult soleus. In contrast, significant increases in BiP, PDI and CHOP were observed in the Old soleus upon reloading.

The pattern of ER stress marker expression in both Adult and Old TA varied slightly from the soleus in response to unloading and reloading. At baseline, levels of BiP and CHOP were elevated in Old TA compared to Adult (Fig. 9A and C). After 14 days of unloading, BiP and PDI increased in Adult TA, while only BiP increased in Old TA. Upon reloading, BiP remained significantly elevated in Old TA and CHOP increased significantly throughout the 14 day reloading period. In general, CHOP levels were significantly elevated in Old, with a greater Adult-Old difference in the TA compared to the soleus (Fig 9C). Overall, these data suggest that ER stress is activated upon reloading of muscles that have previously undergone a period of disuse, but, based on CHOP expression as readout, only in Old does this appear to activate the maladaptive ER stress response.

## DISCUSSION

Disuse causes significant muscle atrophy in both humans and rodents, but this loss in muscle mass can be reversed upon return to normal weight bearing activity.

While aging does not worsen disuse-induced atrophy, it does impact the recovery process, as older individuals do not regain mass or force as quickly as younger individuals [10, 17]. The results of this study clearly show that the recovery of muscle mass and strength after disuse is significantly impaired as a function of age. Further, this study highlights the fact that the effects of aging are highly variable across muscles of varying physiological function and fiber type composition. This is an important point since many studies examine a single muscle and generalize the findings to all muscles.

The mechanisms underlying the adverse effects of aging on the ability of muscle to properly respond to increases in mechanical loading are likely related to multiple factors. Based on previous findings using the functional overload model [19], we initially hypothesized that protein synthesis would be attenuated in the aged rats upon reloading after disuse-induced atrophy. Our data suggests, however, that old rats are able to initiate an anabolic response to reloading. The increase in protein synthesis and ribosome biogenesis is interesting, especially in light of the observation that cage activity is decreased in Old relative to Adult during the first 5 days of reloading. This observation highlights an important unanswered question: Are the same proteins being synthesized in adult and old muscles upon reloading? An increase in protein synthesis is not always associated with a positive outcome, as is the case with denervation [39, 40]. Our targeted gene expression analysis revealed that, at baseline, there is some level of neuromuscular junction remodeling/denervation present in the old muscles, especially the TA, which worsens upon reloading after a period of disuse. These data suggest that neuromuscular transmission is impaired in old muscles, which likely contributes to the poor recovery of muscle mass and strength. In addition, our data points to major deficits in the proteostasis network involved in the proper processing of nascent proteins as well as clearance of dysfunctional proteins, ultimately leading to impaired recovery from disuse-induced atrophy.

### Recovery of muscle mass and strength

A major strength of this study is the examination of multiple hindlimb muscles, which vary in fiber type composition and physiological function, i.e. fast vs. slow and extensors vs. flexors. Hindlimb unloading induces atrophy in all muscles, as shown in the current and previous studies [41], with loss of mass being greater in extensor muscles compared to flexor muscles. Overall, there were no major age-related differences in the atrophy response to 14 days of HU. What was remarkably different between the adult and old rats was the contractile response to the unloading. Following two weeks of unloading, maximum torque decreased by only 17% in Adult, but by 39% in Old. This dramatic drop in force output could not be accounted for by differential loss of muscle mass since adult and old muscles atrophied to the same absolute mass after 14 days of unloading. In this study, the torque measured at the ankle was predominantly an extensor torque given that cutting of the dorsiflexor tendons reduced maximal torque by only 16-19% in Adult and Old (data not shown). A decrease in the specific tension of type I fibers in the MG of 30 month old FBN rats has been reported following unloading [42]. Given the fiber type composition of the extensor muscles and the large decrease in force output, it is likely that the drop in specific force measured in this study reflects a decrease in the specific force of both type I and II fibers. The mechanisms for the loss of specific tension are unknown, but could be related to multiple factors including a change in the stoichiometry between myosin and actin, a decrease in excitation-contraction coupling, or a decrease in force transmission [43].

In contrast to the atrophy response, recovery of muscle mass during reloading differed markedly between the adult and old rats. The impaired recovery of muscle mass in Old was not surprising given previous studies [17, 20]; however, the finding that isometric extensor torque did not significantly improve at frequencies above 60 Hz in Old after 14 days of reloading, even though there were improvements in muscle mass, was unexpected. The delayed improvement of muscle force production in the old rats could be related to one or more of the aforementioned factors; however, our gene expression data suggests that deficits in neuromuscular transmission (i.e. partial or complete fiber denervation) could be playing a major role.

Targeted analysis of specific genes (myogenin, Runx1, Gadd45a, HDAC4) associated with inactivity and neuromuscular junction denervation revealed an increase in all of these genes at baseline in Old, with further increases upon reloading following hindlimb unloading. In general, expression for all of the inactivity-associated genes, except HDAC4, was higher in the Old TA compared to the soleus. Another set of genes elevated in Old was the acetylcholine receptor subunits (α, γ, δ, ε,) Denervation is thought to increase the expression of the adult AChR subunits (α, δ) and de-repress the embryonic subunit γ through activation of HDAC4 that then increases myogenin via repression of Dach2 [44, 45]. Here, we found that the expression of the adult and embryonic AChR subunits was correlated with the changes and relative increases in the expression of myogenin, but not HDAC4. At all-time points in Old, HDAC4 was elevated to a similar extent in both the soleus and TA, while myogenin and the AChR subunits were elevated many fold higher in the TA compared to the soleus. Overall, the gene expression data for Old suggests a greater level of inactivity and/or denervation in the TA compared to the soleus, which corresponds to the greater deficit in recovery in the TA of Old. It should be pointed out that the E3 ligases, MuRF1 and MAFbx, are also thought to be regulated by myogenin [46], however, the expression patterns of MuRF1 and MAFbx were not elevated to a similar degree and did not follow the same pattern of expression as myogenin.

### Aging and the anabolic response to increased loading

In humans, the MPS response to certain anabolic stimuli becomes blunted with age [47, 48]; however, in this study, the old rats did not appear to exhibit impaired activation of ribosome biogenesis or MPS in response to reloading. In fact, increases in MPS were found to occur earlier during the reloading period in Old versus Adult. In both the soleus and TA, MPS was significantly elevated above baseline after seven days of reloading in Old, whereas significant increases above baseline were only seen in Adult after 14 day of reloading, and this only occurred in the soleus. For both age groups, the elevated MPS during reloading may be partially explained by the early increase in Akt/mTORC1 signaling. The most robust increase in signaling was seen for S6K1, which exhibited significant increases in phosphorylation/activation in both the soleus and TA muscles of Adult and Old after three days of reloading. Increased S6K1 phosphorylation is widely considered to be a good marker of mTORC1 activation, which underpins muscle hypertrophy [24, 49]. The increase in MPS at earlier time points during reloading in the TA of Old was likely bolstered by an increase in translational capacity. Total RNA/mg muscle was elevated to a greater degree in the TA of Old versus Adult at day seven of reloading. Consistent with the finding that ribosome biogenesis was activated to a greater extent in Old upon reloading, significant increases in c-Myc were found in the Old TA. c-Myc is a transcription factor that binds to ribosomal DNA and induces transcription of ribosomal RNA (rRNA) genes through the recruitment of RNA Polymerase I [50].

Our finding that ribosome biogenesis was not impaired during reloading in the old rats contrasts with a recent study by Kirby *et al*., who reported that reductions in ribosome biogenesis were associated with the blunted synergist ablation-induced hypertrophy in old mice [27]; however, between-study differences may be explained by differences in the experimental models that were used. Specifically, in comparison to reloading, in which all of the extensor muscles are present, synergist ablation causes the PLN muscle to become the lone load-bearing muscle of the hindlimb, resulting in not only a greater load on the muscle, but also a shift in recruitment, fiber type, and a significantly faster rate of growth. Since mitochondrial content and function has been shown to decrease with age [51], older muscles may not be able to increase energy production enough to meet the high metabolic demands induced by synergist ablation, which could compromise the synthesis of new ribosomes. Thus, the difference in the degree of the hypertrophic stimulus may explain why ribosome biogenesis is impaired with synergist ablation, but not following reloading.

### Aging and proteolysis

In general, proteolytic activity is thought to decrease with age [33], but in skeletal muscle, both increases and decreases in activity of the UPS and autophagy pathways have been reported [52-56]. In this study, there was a trend for the mass of the different hindlimb muscles to be lower in the Old compared to the Adult at baseline, but overall, no significant differences in muscle mass were found between the two ages. These data suggest that 28-29 month old rats are not yet sarcopenic, which is in line with previous findings that show significant atrophy of the hindlimb muscles in F344BN rats at 30 months of age [19]. Comparison of our Adult and Old groups revealed no significant difference in proteasome subunit activities at baseline in the soleus, but activity was higher in a few of the proteasome subunits in the TA of Old. In addition, the level of ubiquitinated proteins in Old soleus and TA muscles tended to be higher, suggesting that the aged animals either had higher E3 ubiquitin ligase activity or a delay in the degradation of ubiquitin-tagged proteins when compared to Adult. The fact that expression of MuRF1 and MAFbx, two muscle-specific E3 ubiquitin ligases associated with muscle atrophy, was not elevated in the old control rats suggests that the aged animals were beginning to experience some impairment in the UPS. To support this theory, basal levels of p62, an autophagy-related chaperone protein that can bind to ubiquitinated proteins and deliver them to autophagosomes for degradation by the lysosome [57], were significantly higher in Old muscles. Furthermore, suppression of the UPS has been shown to activate autophagy [58], and measurement of autophagic flux in control rats revealed a significant elevation of autophagy in Old soleus and TA muscles compared to Adult. The higher resting level of autophagy in Old muscle suggests an increase in protein aggregates that could be related to an impaired degradation of ubiquitin-tagged proteins by the UPS. Another potential stimulus for an increase in autophagy could be defects in protein folding, as indicated by an increase in ER stress.

Muscle fiber remodeling during recovery from disuse atrophy requires increases in both protein synthesis and degradation [28-30]. Upon reloading, increases in degradation might be required for the removal of atrophy-related proteins as well as newly synthesized proteins that have not been incorporated into myofibers or were misfolded or damaged within the ER. Failure to maintain protein quality control can lead to cellular dysfunction [59]. The results of this study indicate that aging impacts the response of both the UPS and autophagy pathways to reloading in a muscle-specific manner. In the soleus, a muscle that likely experienced the highest rate of recruitment upon the return to normal weight bearing activity, increases in both proteasome activity and autophagy were observed during the first week of reloading in Adult, but not Old. Conversely, in the TA, a muscle that likely experienced the least amount of recruitment during the reloading period, activity of the UPS and autophagy pathways were maintained at control levels in the Adult, but in Old, proteasome activity significantly decreased relative to control levels. The lack of an increase in autophagy in the TA of the old rats was interesting given the significant increases in LC3B-II and p62 protein levels during the first seven days of reloading. The inability of the old rats to increase or maintain proteasome activity in the soleus and TA during the early days of reloading may have caused these muscles to rely more heavily on autophagy for protein degradation, which could explain the increase in LC3B-II protein levels seen at day three and seven. However, autophagic flux, measured using colchicine [38], suggests that the rate of autophagy in Old is similar to or less than baseline at day seven of reloading. These data suggest that upon reloading, increases in autophagy, to meet ‘increased demand’, may be blocked by the activation of inhibitory pathways. One potential pathway that could inhibit increases in autophagy is mTORC1/Ulk1. In Old TA, phosphorylation of Ulk1 is significantly increased during the reloading period. Ulk1 has been shown to induce autophagy through its interaction with Beclin, but phosphorylation of Ulk1 by mTORC1 can directly inhibit its activity [37]. Considering the strong activation of the mTORC1 pathway during reloading, the old hindlimb muscles may be receiving mixed signals on whether to increase or decrease autophagy during the early stages of recovery, which may cause or at least contribute to their inability to increase autophagy. Interestingly, in Old TA, cathepsin L activity significantly increases after 14 days of reloading and this is matched by a return of LC3B-II protein to control levels, suggesting that the old rats can increase autophagy, but when compared to the adult, this process is delayed.

### Elevated endoplasmic reticulum stress in aging muscle

Newly synthesized proteins are processed, folded, and transported to other parts of the cell through the endoplasmic reticulum (ER). Perturbations in ER function and suppression of the UPS can lead to the accumulation of unfolded proteins and protein aggregates, which results in ER stress and can trigger the unfolded protein response (UPR) [58, 60]. If the UPR fails to restore ER homeostasis, expression of the maladaptive ER stress marker, CHOP, is upregulated, which promotes cell death through activation of apoptotic processes [61]. We have previously shown that the UPR is activated during the period of elevated MPS in the PLN following synergist ablation [62], and thus we examined the UPR in response to reloading, which is a more physiological growth stimulus.

Surprisingly, at baseline, CHOP protein levels were elevated in both the soleus and TA of Old. This elevation in CHOP expression at baseline could be related to neuromuscular junction remodeling and partial denervation, as increases in CHOP expression are found in denervated muscle [63]. During reloading, the chaperone proteins BiP and PDI increased in both Adult and Old, indicating activation of the UPR. The major difference between age groups was the lack of an increase in CHOP expression in Adult, but a significant increase in CHOP expression in Old, especially in the TA. Thus, it is plausible that the high rate of MPS in combination with impaired activation of protein degradation pathways led to increases in ER stress that could not be alleviated by the UPR. Alternatively, increases in ER stress could be related to defects in the processing of newly synthesized proteins. Thus, we hypothesize that impaired muscle recovery in Old was not due to an inability to initiate an anabolic response to the loading stimuli, but rather was the result of an inability to effectively degrade proteins and properly ‘process’ newly translated proteins for incorporation into the muscle fibers.

## MATERIALS AND METHODS

### Animals

The response of hindlimb muscles to unloading and reloading was studied in 9 month (Adult) and 29 month (Old) male Fischer 344-Brown Norway (F344BN) rats obtained from the National Institute of Aging. Rats were allowed to acclimatize in their cages for at least one week prior to testing. All animal procedures were approved by the Institutional Animal Care and Use Committee at the University of California, Davis.

### Hindlimb unloading/reloading

Unloading of the hindlimbs was achieved by tail-suspension as previously described [64]. The rats were attached via a plastic bar to a swivel mounted at the top of the cage, allowing free 360° rotation. The rats were maintained in ∼30° head-down tilt position with their hindlimbs unloaded for a period of 14 days (n=6-7/group). Rats had access to food and water ad libitum. Body weights of the rats were measured prior to suspension and monitored throughout the unloading period. Animals were released from the tail suspension on day 15 at which time the rats were individually housed and allowed unrestricted cage activity for 3, 7, or 14 days (n=6-7/group).

### Force measurements

Prior to unloading, a series of six isometric contractions was used to determine the *in vivo* baseline isometric torque-frequency profile of the ankle plantar flexor muscles (gastrocnemius, plantaris, and soleus). Rats were anesthetized with isoflurane, placed in a supine position, and their left hindfoot secured to a footplate attached to an Aurora Scientific 300B servomotor. The left leg was clamped in place so that both the knee and ankle were at an angle of 90°. The plantar flexors were stimulated by two needle electrodes inserted proximal to the peroneal nerve.

Torque was measured at stimulation frequencies of 20, 40, 60, 80, 100, and 125 Hz. Contractions were 200 ms, with 45 seconds rest between contractions. Data acquisition and analysis were performed using Dynamic Muscle Control and Dynamic Muscle Analysis software. Isometric torque measurements were repeated in the same rats following 14 days of HU, and at 7 and 13 days of reloading. The final contractile testing period was carried out one day prior to sacrifice.

### Cage activity monitoring

Cage activity of adult and old rats was measured prior to HU and during the first five days of reloading using the Home Cage Photobeam Activity System (San Diego Instruments). Each rat was housed individually and activity was tracked throughout the 12 hour dark cycle and for the first hour of the light cycle. Data was analyzed using the Photobeam Activity System software.

### Muscle collection

Following completion of the appropriate unloading or reloading time period, rats were weighed, anesthetized with isoflurane, and the following muscles were excised from both hindlimbs: soleus, medial gastrocnemius (MG), plantaris (PLN), tibialis anterior (TA) and extensor digitorum longus (EDL). The right soleus, PLN, and TA were pinned at resting length and flash frozen in liquid nitrogen cooled isopentane for histological analysis. All other muscles were weighed and frozen in liquid nitrogen for biochemical analyses.

### Protein synthesis measurements

Changes in MPS were measured using the SUrface SEnsing of Translation (SUnSET) method as previously described [65]. Puromycin was dissolved in sterile saline and delivered by i.p. injection (0.02 μmol/g body wt) 30 minutes prior to muscle collection. Puromycin-truncated peptides, reflecting the rate of MPS, were analyzed by Western blot as described below.

### Fiber cross-sectional area

Serial cross-sections (10 μm) were cut from the soleus using a Leica CM 3050S cryostat and stained with laminin (1:1000, Sigma-Aldrich) to determine fiber cross-sectional area (μm^2^). Digital images were taken under 200X total magnification using an AxioImager M1 microscope. For each muscle, four non-overlapping regions were identified and a total of 100-250 fibers were analyzed within each region using AxioVision software. The same four regions were analyzed across all rats.

### Immunohistochemistry

Immunofluorescence analysis of p62 was performed using 10 μm cross-sections from the TA muscle. Muscle sections were fixed in ice-cold acetone for 10 minutes, washed in phosphate-buffered saline (PBS), blocked with goat serum for 30 minutes, and then incubated with p62 primary antibody (1:500, Sigma) overnight at 4 C. After washing in PBS, the sections were incubated with goat anti-rabbit IgG Alexa Fluor 647 secondary antibody for one hour at room temperature. After a final wash in PBS, sections were mounted with ProLong Diamond Antifade. Digital images were taken under 400X total magnification using an AxioImager M1 microscope.

### RNA isolation and gene expression

Prior to RNA isolation, aliquots of frozen muscle powder from the TA, MG, and soleus were weighed in order to calculate RNA per milligram of wet muscle tissue. RNA was isolated using RNAzol RT reagent according to the manufacturer's instructions and quantified by absorbance spectrophotometry. cDNA was synthesized using a reverse transcription kit from 1 μg of total RNA. Gene expression was analyzed by quantitative PCR (qPCR) using SYBR Green JumpStart *Taq* ReadyMix on an ABI 7900HT thermocycler. Each sample was run in triplicate. Primer sequences are listed in Supplemental Table S1. Gene expression was normalized to tissue weight as previously described [14].

### Western blotting

Frozen TA, MG, and soleus muscles were homogenized in sucrose lysis buffer (50 mM Tris pH 7.5, 250 mM sucrose, 1 mM EDTA, 1 mM EGTA, 1% Triton X 100, 50 mM NaF). The supernatant was collected following centrifugation at 8,000 *g* for 10 minutes and protein concentrations were determined using the Bradford method. Ten to twenty micrograms of protein was subjected to SDS-PAGE on 4-20% Criterion TGX stain-free gels and transferred to polyvinylidene diflouride membrane. Membranes were blocked in 3% nonfat milk in Tris-buffered saline with 0.1% Tween-20 added for one hour and then probed with primary antibody overnight at 4°C. Membranes were washed and incubated with HRP-conjugated secondary antibodies at 1:5,000 (Puromycin) or 1:10,000 for one hour at room temperature. Immobilon Western Chemiluminescent HRP substrate was then applied to the membranes prior to image acquisition. Image acquisition and band quantification were performed using the ChemiDoc™ MP System and Image Lab 5.0 software, respectively. Total protein staining of the membrane was used as the normalization control for all blots. The following antibodies were used: Puromycin (1:500, EMD Millipore), phospho- and total Akt, phospho- and total GSK3β, phospho- and total S6K1, phospho- and total 4EBP1, Ubiquitin, phospho- and total Ulk1, Atg7, Beclin, PDI, CHOP (1:1000, Cell Signaling), p62 (1:2000, Sigma), LC3B (1:1000, Sigma) and BiP (1:1000, BD Biosciences).

### Proteasome activity

20S and 26S proteasome activities were performed as previously described [66]. The caspase-like (β1), trypsin-like (β2), and chymotrypsin-like (β5) activity assays were carried out in a total volume of 100 μl in 96 well opaque plates. The 26S ATP-dependent assays were performed in homogenization buffer with the addition of 100 μM ATP. The 20S ATP-independent assays were carried out in assay buffer containing 25 mM HEPES, 0.5 mM EDTA, and 0.001% SDS (pH 7.5). Proteasome activities were determined by adding 100 μM of Z-Leu-Leu-Glu-7-AMC (Peptide Institute), Boc-Leu-Ser-Thr-Arg-7-AMC (Bachem), or succinyl-Leu-Leu-Val-Tyr-7-amido-4-methylcoumarin (LLVY-AMC) (Bachem) for the β1, β2, and β5 subunits respectively. Each assay was conducted in the absence and presence of the proteasome inhibitor Bortezomib at a final concentration of 2 mM (β5) or 10 mM (β1 and β2). The activity of the 20S and 26S proteasome was measured by calculating the difference between fluorescence units recorded with or without the inhibitor in the reaction medium. Released AMC was measured by a Fluoroskan Ascent fluorometer (Thermo Electron) at an excitation wavelength of 390 nm and an emission wavelength of 460 nm. Fluorescence was measured at 15 minute intervals for 2 hours.

### Cathepsin L activity

Cathepsin L enzymatic activity was assayed by adding 100 μM Z-Phe-Arg-AMC (Calbiochem) to a reaction buffer containing 100 mM sodium acetate, pH 5.5, 1 mM EDTA, and 2 mM DTT in the absence and presence of 10 μM cathepsin L inhibitor I (Calbiochem). Each assay was conducted using 34 μg of protein per well. Fluorescence was measured at excitation and emission wavelengths of 390 nm and 460 nm as carried out for the proteasome assays. Activity was calculated by determining the difference between fluorescence units recorded with or without the inhibitor in the reaction medium.

### Measurement of autophagic flux

Autophagic flux was measured in adult and old rats at baseline and after 7 days of reloading using the autophagy inhibitor colchicine as previously described [38]. Colchicine was delivered by i.p. injection (0.4 mg/kg/day) for two days prior to muscle collection.

### Statistics

Results are presented as mean ± standard error of the mean (SEM). The difference in starting body weight between adult and old rats was analyzed by Student's *t* test while the remaining data was analyzed by one- or two-way ANOVA using GraphPad Prism software (GraphPad Software). Tukey's post hoc analysis was used to determine differences when interactions existed. Statistical significance was set at p<0.05.

## SUPPLEMENTARY MATERIAL TABLE AND FIGURES


