# Supplementary material for: Age-related deficits in skeletal muscle recovery following disuse are associated with neuromuscular junction instability and ER stress, not impaired protein synthesis
Source: Aging (Albany NY). 2016 Jan 29;8(1):127–46. doi: 10.18632/aging.100879 (PMC4761718; doi:10.18632/aging.100879)
Supplement: Supplementary file 1 [file aging-08-127-s001.pdf]

Supplemental Table S1. Rat primer sequences.

Gene	Forward	Reverse
nAChRα	GTCACCCACTTTCCCTTCGA	CCAGATAAGGGGTGTTGGGG
nAChRγ	GCCCATCATGACTCGAGAGG	AGTACTGCCTGCACCAATCC
nAChRδ	GGAGAACAACAATGACGGCTC	TGGCCCGAGTCAGAAACAAG
nAChRε	ATGGCAAGACTATCGGCTCA	AGCCATACATGTTCGGAAGGG
c-Myc	CAGCAGCGACTCTGAAGAAGAAC	GATGACCCTGACTCGGACCTC
Gadd45a	GTCACTCCCCACGCTGATG	TGCAAAGTCATCTCCTAGCCC
HDAC4	AGAGGTGAAGATGAAGCTGCA	GCTGTGTCTTCCCATACCAGT
ITS-1	TCCGTTTTCTCGCTCTTCCC	CCGGAGAGATCACGTACCAC
MAFbx	CATCCTTATGCACGCTGGTC	GGTCTCCATTCGATACACCCA
MuRF1	ACAACCTCTGCCGGAAGTGT	CCGCGGTTGGTCCAGTAG
Myogenin	CCAGGAGATCATTTGCTCGCA	TGGGCATGGTTTCATCTGGG
Runx1	ACCTACTCGCCTCCTGTCA	AGGTAGGTGTGGTAGCGAGAG

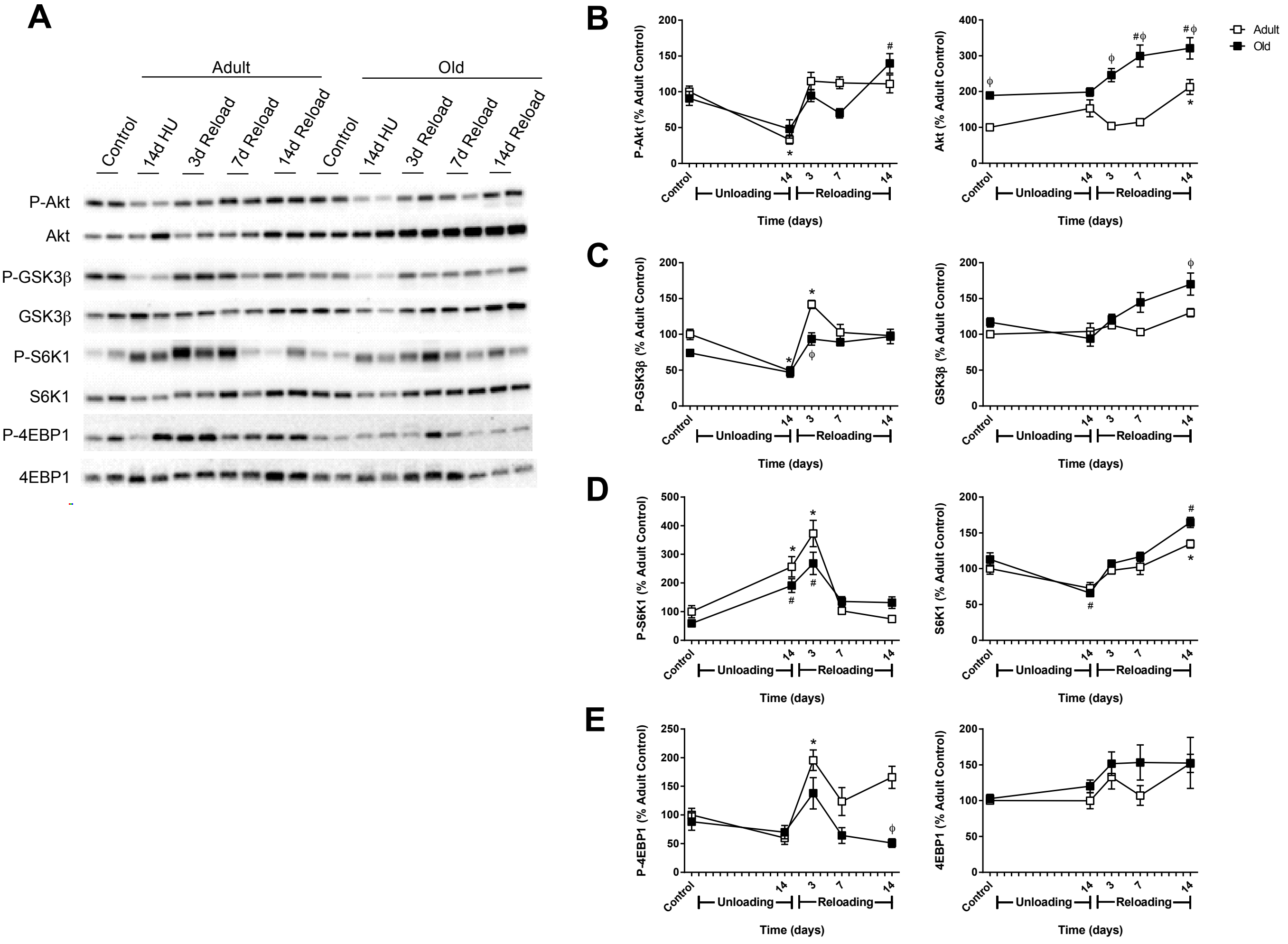

Supplemental Figure 1. Akt/mTORC1 signaling in the soleus muscle of adult and old rats during hindlimb unloading (HU) and reloading. Representative Western blots **(A)** and quantification of phospho- and total Akt **(B)**, phospho and total GSK3β **(C)**, phospho- and total S6K1 **(D)**, and phospho- and total 4EBP1 **(E)** protein expression in the soleus muscle of adult (9 mo, open squares) and old (29 mo, filled squares) rats after 14 days of HU and following 3, 7, and 14 days of reloading. Total protein, determined by stain-free imaging of the PVDF membrane, was used to normalize protein expression. Data are expressed as a percentage relative to the adult control group for each protein (n=4-6/group). Values are mean ± SEM, *$p<0.05$ vs adult control, $^{\#}p<0.05$ vs old control, $^{\varphi}p<0.05$ vs adult at same time point.

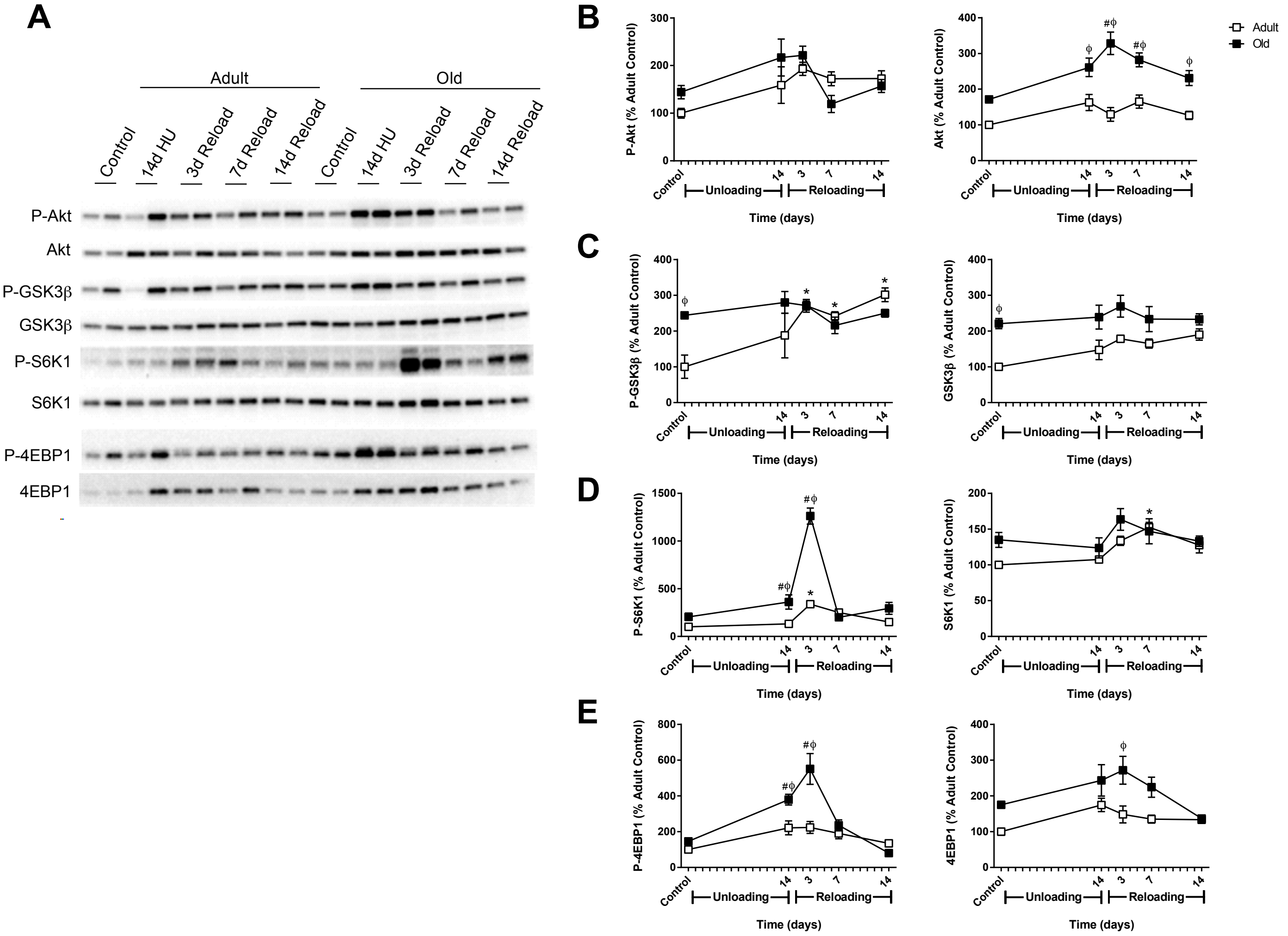

Supplemental Figure 2. Akt/mTORC1 signaling in the tibialis anterior (TA) muscle of adult and old rats during hindlimb unloading (HU) and reloading. Representative Western blots **(A)** and quantification of phospho- and total Akt **(B)**, phospho and total GSK3β **(C)**, phospho- and total S6K1 **(D)**, and phospho- and total 4EBP1 **(E)** protein expression in the TA muscle of adult (9 mo, open squares) and old (29 mo, filled squares) rats after 14 days of HU and following 3, 7, and 14 days of reloading. Total protein, determined by stain-free imaging of the PVDF membrane, was used to normalize protein expression. Data are expressed as a percentage relative to the adult control group for each protein (n=4-6/group). Values are mean ± SEM, $^{*}p<0.05$ vs adult control, $^{\#}p<0.05$ vs old control, $^{\varphi}p<0.05$ vs adult at same time point.

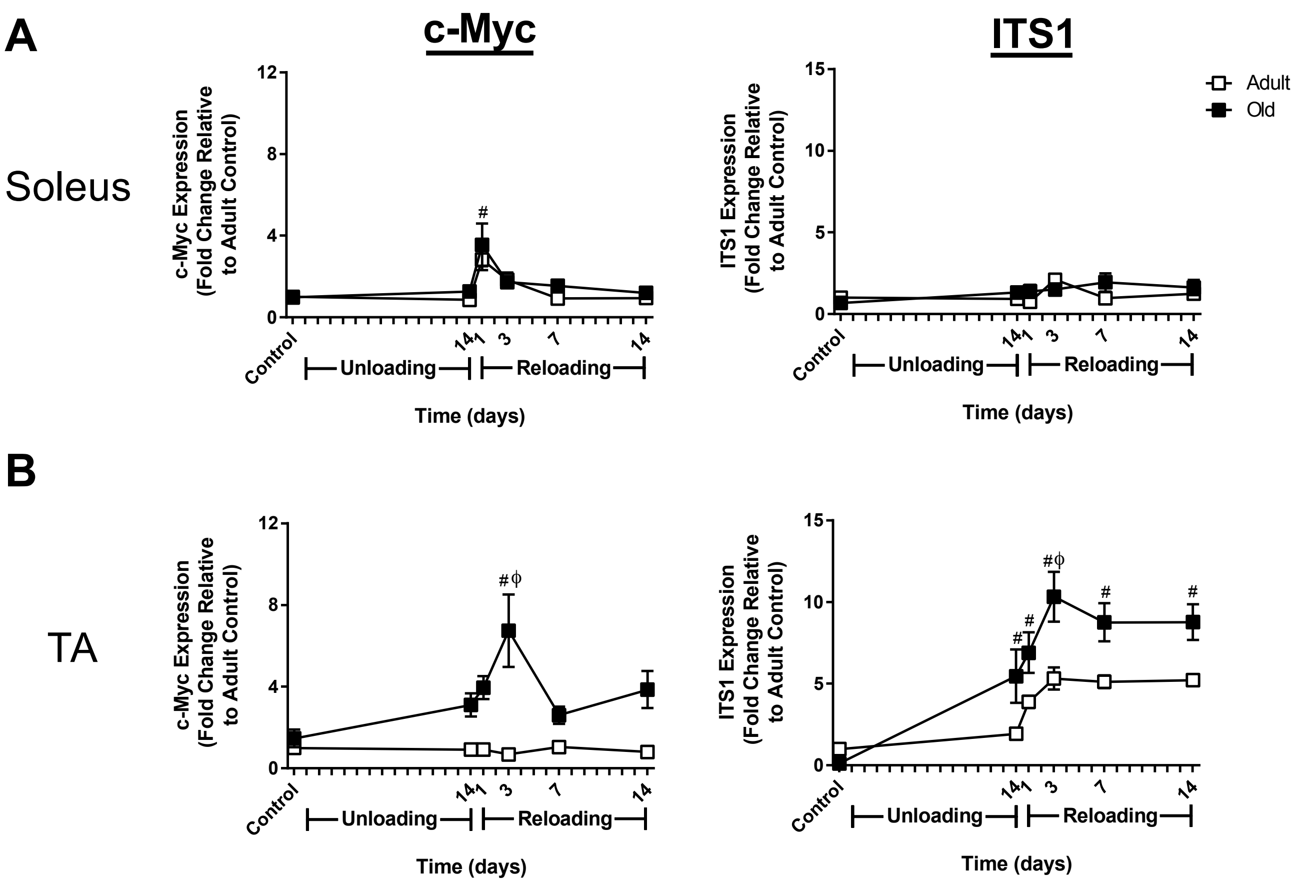

Supplemental Figure 3. c-Myc and ITS-1 expression changes in the soleus and tibialis anterior (TA) muscles in response to hindlimb unloading (HU) and reloading in adult and old rats. mRNA expression (fold change relative to adult control) of c-Myc and ITS1 were measured in the soleus **(A)** and TA **(B)** muscles of adult (9 mo, open squares) and old (29 mo, filled squares) rats after 14 days of HU and after 1, 3, 7, and 14 days of reloading (n=6/group). Values are mean ± SEM, $^{*}p<0.05$ vs adult control, $^{\#}p<0.05$ vs old control, $^{\varphi}p<0.05$ vs adult at same time point.

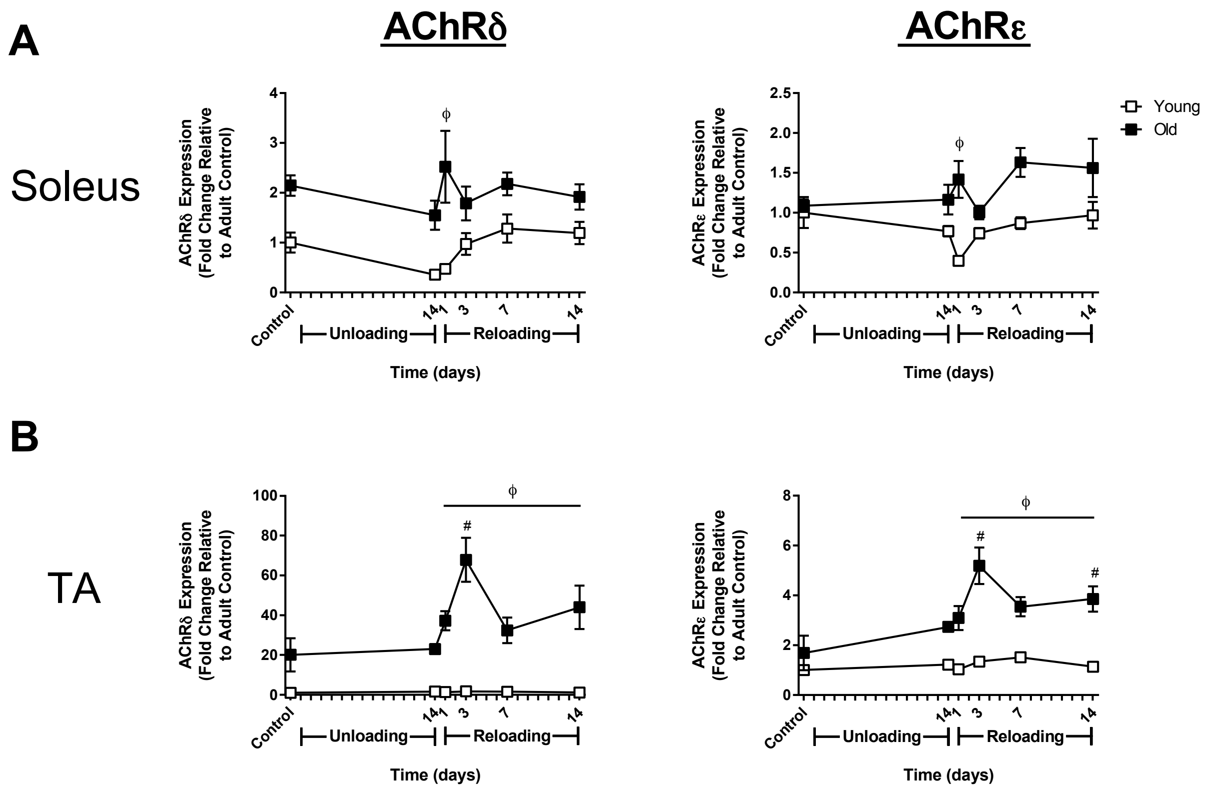

Supplemental Figure 4. Expression changes of the acetylcholine receptor subunits delta (AChRδ) and epsilon (AChRε) in the soleus and tibialis anterior (TA) muscles in response to hindlimb unloading (HU) and reloading in adult and old rats. mRNA expression (fold change relative to adult control) of AChRδ and AChRε were measured in the soleus **(A)** and TA **(B)** muscles of adult (9 mo, open squares) and old (29 mo, filled squares) rats after 14 days of HU and after 1, 3, 7, and 14 days of reloading (n=5-6/group). Values are mean ± SEM, *$p<0.05$ vs adult control, $^{\#}p<0.05$ vs old control, $^{\varphi}p<0.05$ vs adult at same time point.

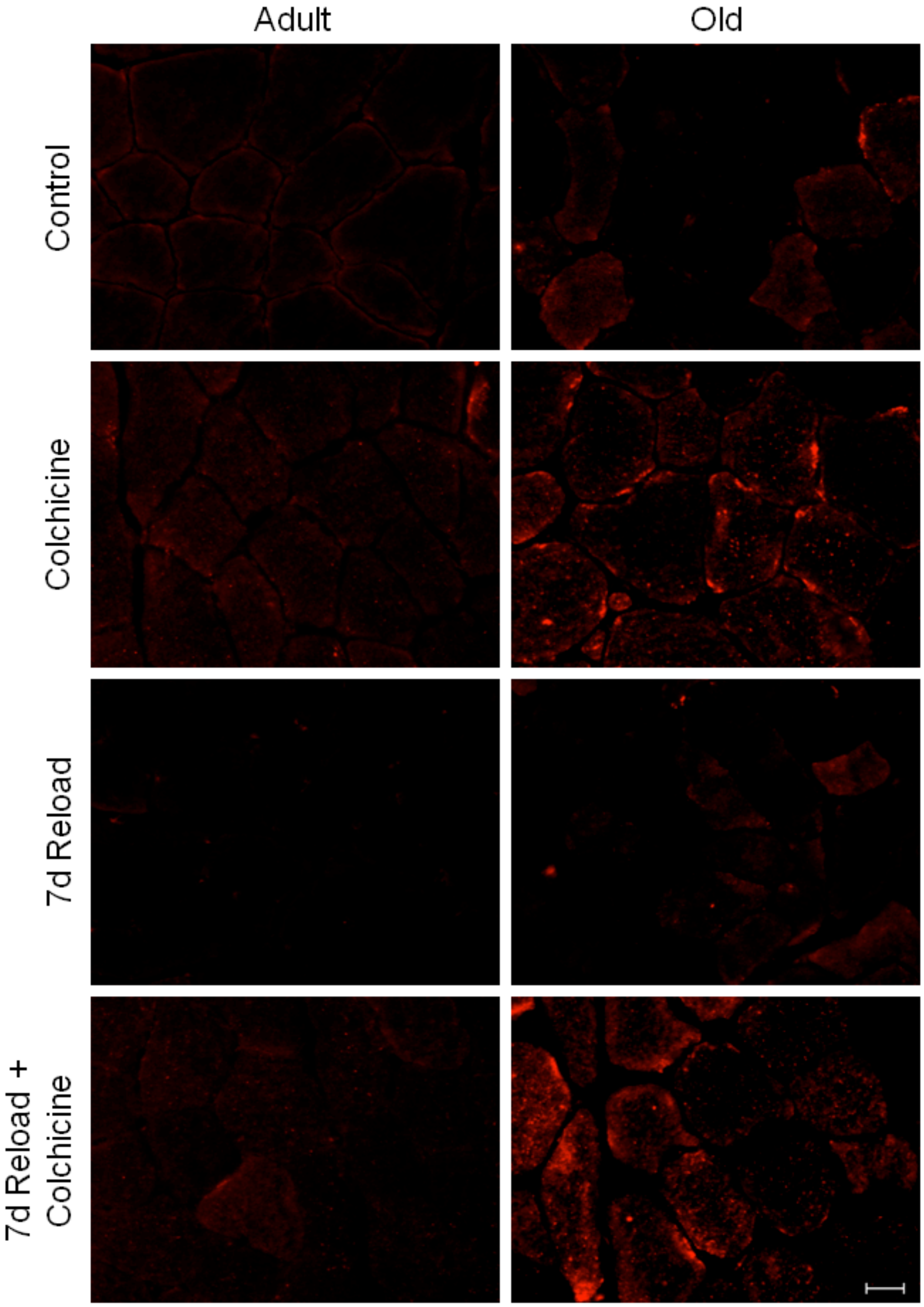

Supplemental Figure 5. Effect of age and reloading on p62 protein accumulation using the autophagy inhibitor colchicine. Immunofluorescent staining of p62 in the TA muscle of adult and old control and 7d reloaded rats treated with or without colchicine. Scale bar: 20 μm
